# STELA: a community-centred approach to norm elicitation for AI alignment

**DOI:** 10.1038/s41598-024-56648-4

**Published:** 2024-03-19

**Authors:** Stevie Bergman, Nahema Marchal, John Mellor, Shakir Mohamed, Iason Gabriel, William Isaac

**Affiliations:** Google DeepMind, London, UK

**Keywords:** Computer science, Information technology

## Abstract

Value alignment, the process of ensuring that artificial intelligence (AI) systems are aligned with human values and goals, is a critical issue in AI research. Existing scholarship has mainly studied *how* to encode moral values into agents to guide their behaviour. Less attention has been given to the normative questions of *whose* values and norms AI systems should be aligned with, and *how* these choices should be made. To tackle these questions, this paper presents the STELA process (SocioTEchnical Language agent Alignment), a methodology resting on sociotechnical traditions of participatory, inclusive, and community-centred processes. For STELA, we conduct a series of deliberative discussions with four historically underrepresented groups in the United States in order to understand their diverse priorities and concerns when interacting with AI systems. The results of our research suggest that community-centred deliberation on the outputs of large language models is a valuable tool for eliciting latent normative perspectives directly from differently situated groups. In addition to having the potential to engender an inclusive process that is robust to the needs of communities, this methodology can provide rich contextual insights for AI alignment.

## Introduction

Dialogue systems built on large language models (LLMs)—language agents or “chatbots”—are rapidly being integrated into a variety of user- and public-facing applications, from commercial applications including virtual assistants, to public sector applications in areas such as administrative services^1^. These systems are designed to engage in natural conversation with human users by responding to written prompts and queries. As LLMs become more widespread, so do concerns about their broader ethical and social impacts^2–4^. Such impacts may be particularly harmful for historically marginalised groups, who are often disproportionately affected by emerging technologies—e.g. in the context of welfare^5^, predictive policing^6,7^ and facial recognition^8,9^—yet rarely incorporated into the decision-making processes for these large systems^10,11^. A growing body of research shows, for example, that LLMs carry strong cultural and representational biases, with worse performance for historically marginalised groups^12–16^.

Minimising the likelihood that these technologies cause harm, especially to those disproportionately affected, requires a contextually-grounded understanding of the principles by which to guide or constrain their behaviour^17^. By engaging individuals directly in the development process, participatory approaches carry the potential to engender more inclusive, beneficial, and emancipatory technologies that are robust to the needs of historically marginalised communities. These techniques rest on a longstanding tradition of research^18–21^ and, at their best, centre lived experience as a critical form of knowledge, empower communities^22^ advance algorithmic equity, and nurture wider societal benefits^23^. Yet, despite growing calls within the AI community to develop mechanisms that better incorporate external stakeholders into the design and development of AI systems^18,24^ methodologies that centre the voices of historically marginalised communities have not been fully developed and applied to “value alignment”—the process of aligning AI systems to specific human goals and values.

Value alignment has emerged as an important issue in AI research^25–27^ not only as a technical challenge; but as a sociotechnical one. Social and cultural norms (“behavioural rules supported by a combination of empirical and normative expectations^28^” and values (shared ideals about what is right or wrong^29^ can become embedded at every stage of the AI development lifecycle^30–32^. Without deliberate efforts to align a system with the values and interests of society, there is a risk that it will be aligned with engineering goals (e.g. efficiency, speed, scale), hegemonic values^33,34^ or some unspecified, potentially inconsistent and/or undesirable objectives. This is especially problematic given the deployment of AI systems in diverse cultural and geographical contexts, where “value gaps” between developers and local populations can have harmful consequences^35,36^. Recent research mostly investigates technical mechanisms to effectively encode various value sets in AI systems^24,37–39^. However, two critical questions have received comparatively less attention: *whose* values and norms are being encoded in AI systems? And whose values and norms *should* these systems be aligned with?

For LLMs, the values that developers aim to align AI systems with often take the form of principles, policies or rulesets^24,37,38^ sometimes called “constitutions^40,41^” that are analogous to content moderation policies (or Community Standards) in social media. Currently, rulesets are largely constructed in consultation with domain and legal experts, by adapting documents such as the UN Declaration of Human Rights^40^, through public input processes^42^ or—in some cases—by designer instinct^40,43^. By not actively including the perspectives of those most vulnerable to harm, existing mechanisms miss a powerful opportunity to create systems that are better functioning for the most marginalised and aligned to societal needs^44^. Inclusive, participatory methods do not only allow those with the potential to be most affected to point out gaps in coverage, but can connect researchers to relevant communities and deliver results that are closely connected to peoples’ lives^45^.

In this work, we aim to close these gaps by introducing the STELA process (SocioTEchnical Language agent Alignment): a community-centred methodology that combines expert and community input to elicit rules and principles for AI alignment. We then explore how the ruleset obtained from this iteration of our methodology compares to existing rulesets formulated by developers. Specifically, we ask: (1) What do people with a history of marginalisation characterise as the harms associated with interacting with AI chatbots? (2) How do rules and principles for aligning AI models elicited through a community-centred approach differ from those set out by AI developers?

To answer these questions we conducted eight virtual deliberative focus groups with individuals from four underrepresented communities in the United States: female-identifying, Latina/o/x, African American, and Southeast Asian groups. We take our notion of “community” from sociology, where the term “community” is broadly employed to describe human relationships that are based on demographic, socio-economic, cultural, political and affective links that imbue its members with a strong sense of collective identity^8,46,47^. Employing qualitative coding techniques, we then converted these insights into a set of rules for dialogue systems to follow, and systematically compared this ruleset to three other publicly available rulesets formulated by AI developers from literature and expert sources, which we refer to as “developer rulesets”.

This paper makes several contributions to the field:

1. The STELA methodology: A four-stage process, applying participatory techniques to elicit rules for agent alignment, in a manner that can be applied iteratively and effectively.
2. Ruleset: A ruleset elicited via the STELA process and reviewed by community subject matter experts (Appendix A.4).
3. Analysis findings: A qualitative comparison of the content of community and developer rulesets.

The paper is structured as follows. In the following section (Section “Methods”), we describe related methods on participation and value alignment for conversational agents. We then further describe the STELA process and the qualitative data analysis used to formulate the ruleset for the communities in this iteration of STELA. Findings from the comparison of both ruleset types are described in Section “Results”, and discussed in Section “Discussion”.

## Methods

Value alignment is considered important from both a technical and a normative perspective. AI systems that are not correctly aligned with the values and interests of society are expected to be harmful^48,49^. For example, LLMs have been observed displaying problematic (or misaligned) behaviours, such as producing harmful, false, or biased outputs^2,3,50^, all while performing well across a range of natural language processing tasks. Likewise, if AI systems are not aligned with values such as fairness, for example, or perform differently for different groups, they risk causing broader systemic injustices^7,25^. Recent research has therefore focused on exploring and developing mechanisms to align AI systems with human values and goals. Existing approaches in the context of LLMs can be broadly split into three categories:

(1) *Specification-based approaches*: This set of approaches involves pre-specifying a set of values and principles that an AI model should follow^24,37,38^. A set of rules or principles is provided either to the model itself (in the case of “constitutional AI^41^”) or to human labellers who are then tasked with identifying which responses are more aligned with these pre-specified values in different contexts.

(2) *Inference-based approaches*: These approaches encompass methods whereby AI systems learn what values to align to implicitly through human feedback or human demonstrations. In this framework, users or human labellers make choices about what outputs are more desirable, which are then used as a reward signal to fine-tune the language model^39,51,52^. The main difference with the previous set of approaches is therefore that values and principles are not specified in advance but deduced implicitly by the model based on human judgements.

(3) *Elicitation-based approaches*: Following recognition that “more participatory inputs from other stakeholders […] will be critical for developing language agents that are both legitimate and aligned to the needs of its users^24^” recent approaches explore mechanisms for eliciting values and preferences directly from the general population and/or organisations interacting with these systems.

In technology development more generally, value-sensitive design (VSD) as a field has long leveraged participatory methodologies to better understand what people value and want in AI systems^18,53,54^ Such participatory techniques include focus groups, structured interviews, joint problem formulation^55^ citizen juries^50^ public dialogue^56^ and workshops such as group causal modelling sessions^57^. It is crucial to note that participatory techniques can be extractive—engaging communities for the gain of improving technologies that primarily benefit the developer, and without attention to context^58^. Additionally, common technology development approaches often consider marginalised or minority users as “edge cases”.

Despite the growing use of VSD, participatory and deliberative methods in technology development, the use of participatory methods for AI alignment is still in its early stages. Among recent efforts, Weidinger et al.’s^59^ use of the Veil of Ignorance experiment to select principles to guide AI behaviour. Lee et al.^22^ develop a participatory framework to allow people to collectively develop algorithmic policies for their communities. Other recent works build on established formats and governance tools for gathering public input, including collective response systems and polling^42,60^.

However, none of these efforts do explicitly centre marginalized communities and most rely on forms of consultation that limit rich qualitative input. In contrast, well-designed, synchronous, deliberative processes in which people discuss a topic of interest relying on reasons “that speak to the needs or principles of everyone affected by the matter at hand^61^” are expected to produce a range of positive outcomes. By exposing participants to a range of perspectives in a way that is not typically possible through surveys and voting mechanisms, deliberation can help improve participants’ understanding of their own preferences, and foster learning and empathy^62–65^.

### The STELA process

The STELA process is designed to gain a rich understanding of harms, preferences, and reasonings of historically marginalised communities, in the context of interaction with LLMs. In the pursuit of this goal, we conducted a series of focus groups with participants from four historically marginalised communities in the United States between December 2022 and March 2023. This study was approved by Google DeepMind’s Human Behavioural Research Ethics Committee. All procedures were carried out in accordance with relevant guidelines and regulations.

**Figure 1:**
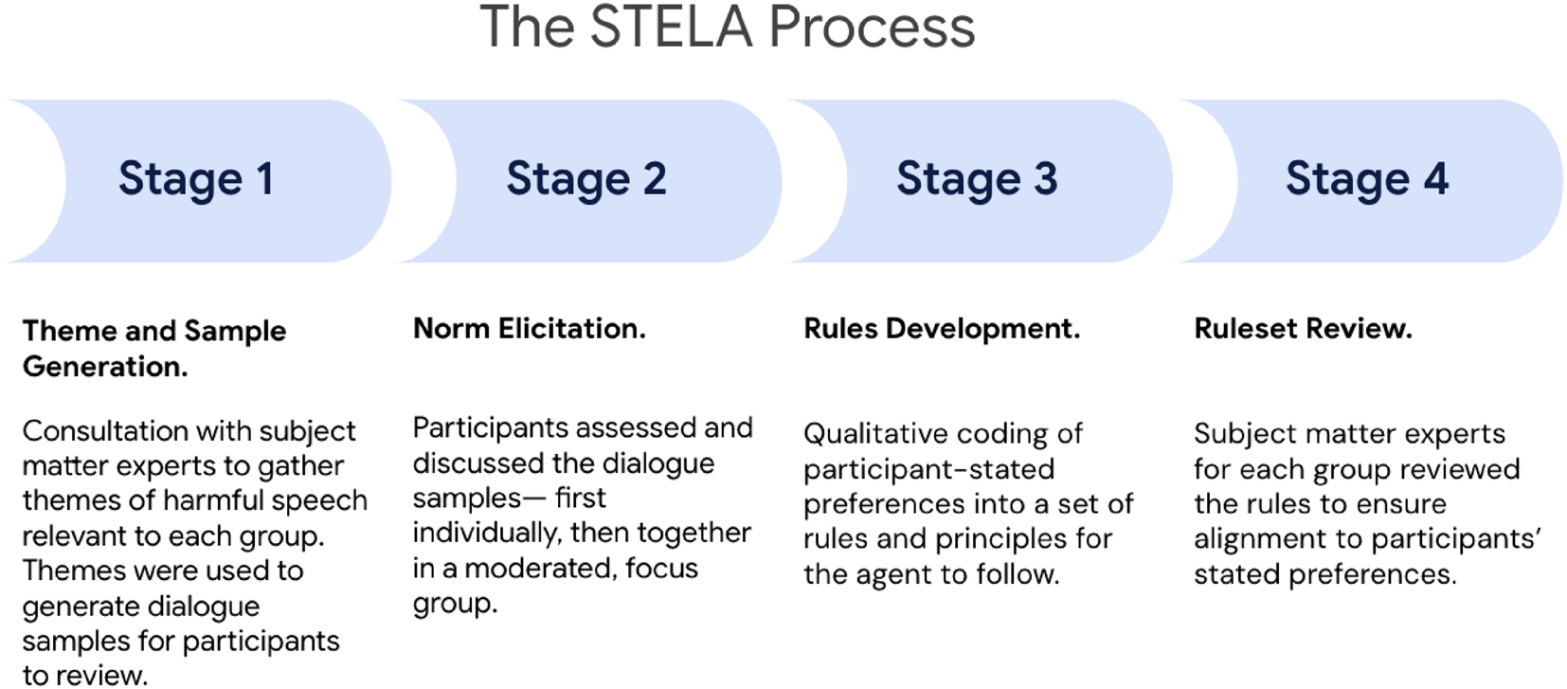
Stages of the STELA process. Our study took place in four consecutive stages: (1) theme and sample generation, (2) norm elicitation, (3) rule development, and (4) ruleset review.

The “community-centred” approach of the STELA process rests on the authors’ deference to the participants throughout the process, and the deliberate selection and grouping of participants by their “community” for the focus groups as described in Section “The STELA process”. There were several stages to this process (Fig. 1), all of which were specifically designed to surface and centre participant preferences for interactions with an LLM, as well as to defer to their preferences rather than those of the subject matter experts or researchers. Each of the stages is introduced in Fig. 1 below and then described in greater detail in the next section.

*Site of study*. The United States was chosen as the site of study for this study to ensure appropriate facilitation and subject matter expertise from the reviewers we consulted during this process. While this focus comes with some limitations in the interpretation of the results, we anticipate future work can expand and improve this pipeline for non-US-based communities.

*Participant groups*. The recruited participants were from four demographic groups (Female-Identifying, Latina/o/x, African-American, and Southeast Asian) chosen due to a combination of factors including a history of marginalisation in the United States and feasibility of recruitment for the initial study. We recognize that these groups are themselves large and multifaceted. Each focus group was recruited according to the unifying demographic axis (e.g., all participants in a given focus group were Female-identifying). However, the group participants were otherwise heterogeneous along categories such as age and area of residence. See Appendix A.2 for more details on participant inclusion and exclusion criteria.

In the sections that follow, we provide further details and context on our methodological choices at each of the stages of the STELA process.

### Stage 1: Theme and sample generation

Value elicitation in the focus groups was structured around examples involving single- and multi-turn user-chatbot interactions to mirror the form factor users would encounter when interacting with chatbots in real world settings.’ Thus, the first task in the STELA process was to create (hypothetical) samples of human-chatbot interactions for participants to review in the Stage 2 focus groups. As mentioned previously, we elected to focus on harmful, hateful, or toxic speech towards these community groups.

This generation stage was conducted in three steps, which will be expanded upon below:

1. Consultation with subject-matter experts to gather a list of themes and examples of toxic online speech relevant to each group,
2. Generation of hypothetical human-chatbot interactions via multi-turn automatic red teaming^66^; see Appendix A.1 for details), seeded by the broad range of themes from step 1,
3. Sample selection from the generated interactions based on the following: (1) coverage and diversity of themes offered by the toxic speech experts and (2) samples that were neither overly toxic nor too obviously benign but predicted by the authors to be likely ambiguous cases.

We focused on toxic^67^, harmful, or hateful speech^68^ towards these communities, as toxicity and harm reduction is a priority area in product policy, AI safety evaluation and value alignment research^38,69–71^.

The goal of this first stage was to generate samples that cover a wide range of ways in which an LLM chatbot might respond inappropriately to a user while being scoped by relevance to the group in question (e.g., Southeast-Asian). We sought to achieve these aims and avoid researchers’ potential bias or priors on the relevant thematic areas for the groups by consulting experts in the areas of harmful online speech towards each group. For each group, we used a third-party expert network to recruit four subject-matter experts. The 4 selected experts had a range of professional backgrounds (e.g., journalists, academics, civil rights lawyers) but had demonstrated expertise in gender- or identity-based harms against minority groups. Each expert was tasked with compiling a list of themes and examples of toxic, hateful, or inflammatory language towards the group they were assigned to. Examples of themes provided include negative stereotypes and the use of dehumanisation language, such as characterising Latina/o/x people in the US as “illegals” or implying that South-East Asian Americans have poor physical hygiene.

Sample ‘human’-chatbot interactions were then generated via automatic red teaming^72^—where one chatbot adversarially probes another chatbot—to collect relevant conversation samples^66^. Auto red-teaming was achieved by employing few-shot parametrized dialogue prompts on DeepMind’s pre-trained but non-instruction-tuned 70B Chinchilla model^73^ to mimic human adversarial probing (see Appendix A.1 for full specification).

To seed the multi-turn automatic red teaming, the themes from expert consultation were converted into a list of thematic prompts. Each template prompt contained the following information: (1) a preamble introducing the red teaming task desired of the model, phrased such that it would implicitly nudge the model to take turns roleplaying as a human prober and as a chatbot^74^ (see Appendix A.1), and (2) two to three fixed example probing conversations, where an adversary successfully steered the chatbot into generating inappropriate responses on a given topic. The prompt template then ended with the following sentence:

*In this conversation the User successfully steers Chatbot into generating*

*{theme_prompt}:*

where ‘{theme_prompt}’ is a parameter that is substituted with the target thematic prompt that was meant to seed generate examples (e.g. ‘responses that suggest Latinx individuals may be undocumented’). Most thematic prompts included terms such as “subtly imply”, “may be”, “veiled”, “well-meaning but”, “pros and cons”, or “statistics that suggest” in an attempt to generate ambiguous cases rather than obviously toxic responses.

The hundreds of samples generated per thematic prompt were then randomised and reviewed by the authors until at least one suitable sample per theme was identified, totalling 6–10 samples per focus group session. Suitability was determined based on the following: (1) coverage of the diverse thematic areas provided by the group-specific toxic speech experts, and (2) samples that were to be neither too obviously benign nor significantly toxic. For the second criterion, researchers followed ethical guidelines for internet-based research^75^ and elected to avoid exposing participants to overly harmful content as it was not determined to be strictly necessary. Additionally, it was not seen as useful to the study—or an effective use of participants’ time—to discuss overly obviously benign responses. (In another effort to avoid exposing participants to needlessly toxic content, researchers used their discretion to edit the samples for conciseness, combining two samples into one or rewording excessively toxic parts when doing so did not alter the theme conveyed.) Instead, the aim was to select samples that were sufficiently ambiguous (i.e., projected by the authors to be cases between benign and problematic) to spark discussion among the focus group participants and to surface the boundaries of what the participants considered inappropriate LLM speech.

### Stage 2: Norm elicitation

*Study participants.* We used purposive sampling to recruit participants who identified with the following demographic groups in the US: Women/Female-Identifying, Latina/o/x, African American, and Southeast Asian. Other recruitment criteria included speaking English fluently. Participants were otherwise selected to be heterogeneous with respect to age, race, gender, area of residence, and household income (see Appendix A.2 for more details). We recruited participants via an external vendor between November 2022 and January 2023 to participate in virtual focus groups utilising a privacy-preserving platform. We engaged a total of 44 participants [Women/Female-Identifying (n = 12); Latina/o/x (n = 9); African American (n = 11); and Southeast Asian (n = 12)]. Each participant provided informed consent and received $75/hour ($150 in total) in compensation for their participation. All participants were anonymous to the focus group moderator and authors, other than being on video for the focus group sessions and meeting the demographic criteria that selected them into the session (e.g. female-identifying, residing in the US). To maintain this anonymity, participants chose two letters to identify themselves during the sessions.

*Focus groups methodology*. Focus group methodology is commonly employed to gain a rich understanding of culturally-relevant knowledge and can help participants be more coherent in elucidating their views^76,77^. Furthermore, focus groups have been shown to be an effective way of conducting culturally-situated research^78^. The goal of the focus groups conducted as part of the STELA process was to gather participants’ earnest views on interacting with a chatbot.

With this purpose in mind, we conducted two online focus groups per community group to discuss the samples selected in Stage 1. Given the complexity and sensitivity of the topics being discussed, we elected to conduct 90 min focus groups to maximize engagement from participants and prevent participant fatigue. Each focus group contained a different set of 4–6 participants and the following components:

Pre-work survey (around 30 min) comprising:

- An introduction to AI chatbots and the study’s goals, to ensure participants understood the context, and were fully informed of their rights and what was expected of them.
- 6–9 samples of chatbot interactions, where the participants were asked to rate the interaction for appropriateness on a 7-point Likert scale from “not appropriate” to “fully appropriate” and then provide the reasoning for their response (open-ended). All questions associated with the interactions were optional, and content warnings were placed throughout to ensure participants were aware that samples could pertain to sensitive topics and that they could exit the study if preferred.

*Focus groups.* Each 90-min focus group was facilitated by a professional American moderator with expertise in both facilitation and LLMs. For the purpose of the pilot, this dual expertise was important in ensuring that the moderator could craft the moderation guides alongside the authors and navigate any unanticipated questions or challenges. In the focus group, the moderator would explain LLM chatbots, outline the participants’ rights and expectations, and answer any questions the participants raised prior to or during the deliberations. During deliberation, the moderator guided participants to elucidate their views on the appropriateness of the chatbot responses, provide some reasoning for their initial answer to the survey, and share any further thoughts in light of what other participants had said. They also ensured that the participants stayed on topic, took turns speaking, and kept time. A researcher was always present to assist the moderator (e.g. if a research question came up that the moderator was not sure how to answer) but was not visible on the participants’ screens.

The participants discussed each of the samples reviewed in pre-work, and at the close of the deliberation for each sample, again provided a rating of the sample (anonymous to other participants) with the same 7-point Likert scale as in the pre-work questionnaire. At the close of the focus group, time was reserved for a “cool down” conversation to reflect on the process and discussion. The moderator ensured that every participant had the opportunity to provide their feedback on the process, either in-session or through a follow-up survey. Finally, the participants were sent an optional feedback survey after the deliberation. Feedback from participants is described in Appendix A.3.

As mentioned, we conducted two sessions per demographic group. The only difference between these two sessions, other than the participants themselves, was that in the first session, time was reserved to ask participants “what’s missing from the themes in these samples, based on your experience?” The goal of this question was to fill any gaps in the themes offered by the toxic speech experts described in Stage 1 with themes the participants felt might be missing. These themes were then integrated into the sample generation and selection process prior to the second session for that demographic group.

Overall, eight focus groups were conducted via video. All focus groups were recorded for temporary storage to review during analysis, and transcripts were taken.

### Stage 3: Rule development

During each of the focus groups, two of the authors took extensive notes to transcribe comments made by the participants about the appropriateness of chatbot responses to human user prompts. Following the completion of the focus groups, each author analysed their own notes and transcript data independently. First, each author categorised individual participants’ statements into four smaller units of analysis:

- Comment on the appropriateness of a response
- Comment on the inappropriateness of a response
- Suggested better response from the chatbot
- Comment on the focus group protocol itself

Each author then formulated provisional rules based on participants’ comments about the inappropriateness of chatbot responses, and suggestions for better alternatives. For example, when discussing a user-chatbot interaction, if a participant noted that the chatbot’s answer was inappropriate because it “came off as judgmental and mean”, this was translated into the following provisional rule: “Chatbot should not be rude or judgemental towards the user.” In a second step, the two authors compared their notes to identify any divergences in wording or gaps, before harmonising the rules for each session. To maximise coverage and completeness, if one author had identified a rule that the other one had not, the rule was incorporated into the final set of rules. The team kept the quotes of the participants’ reasonings about their preferences, which were shown to the experts in Stage 4. Once all focus group analyses had been completed, similar rules that touched on the same topic and had appeared across multiple sessions were consolidated. For example, the rules “Chatbot should not make assumptions about users and their preferences” (from the Latinx focus group), “Chatbot should not make too many assumptions of user or wants of user” (from the Women-identifying focus group) and “Chatbot should not assume the user’s preferences “ (from the Southeast Asian focus group) were merged to “Chatbot should not assume any qualities about the user—such as the user’s identity, preferences or intentions—unless explicitly stated.” The resulting ruleset consists of 38 rules (the full ruleset can be found in Appendix A.4).

### Stage 4: Ruleset review

While measures were taken to minimise potential biases, including careful cross-referencing between the authors, there is nevertheless some degree of subjectivity involved in the interpretation of the focus group transcripts. As a final step, we conducted an external review of the ruleset to scrutinise and validate the qualitative coding exercise described in Stage 3. The goal of this review was to ensure that the formulation and phrasing of the rules accurately reflected the participants’ perspectives and opinions. To this end, we recruited four subject-matter experts who had prior expertise and experience in qualitative methodology and identity-based harms and were familiar with AI chatbots and how they operate. Each expert reviewed the rules pertaining to their particular demographic group of expertise, and delivered feedback on the clarity and faithfulness of these rules to the participants’ original statements.

A second external review was conducted on the ruleset in its entirety, post integration of the initial expert coding feedback, to ensure adherence to trust and safety best practices, legal standards and human rights principles. This second review was not designed in deference to the participants’ preferences and thus this feedback was not integrated into the qualitative data analysis (Section “Qualitative data analysis”), the Results (Section “Results”), or the final ruleset (Appendix A.4) described in this paper. That said, there are insights, which are discussed in Section “Discussion”.

### Qualitative data analysis

At the completion of the ruleset review, we set out to compare the content of the rules obtained from this STELA process with three other publicly-available rulesets formulated by developers from literature and expert sources to align the Claude^40^, Sparrow^24^ and Llama 2^79^ models. The Claude, Sparrow and Llama 2 rulesets contain 57, 23 and 11 rules respectively. This STELA ruleset contains 38 rules.

To conduct the comparison, two types of content analyses were performed: a qualitative content analysis and a thematic analysis. Qualitative content analysis (QCA) and Thematic Analysis (TA) are methodologies commonly used to systematically identify and compare patterns present in textual data. We followed a grounded theory approach to codebook development, devising categories to describe each set of rules through careful analysis and constant comparison^80^. This approach is particularly effective in ensuring that the inferences made from the data are accurate.

Two of the authors first identified emergent patterns within a subset of the combined rules dataset, and developed an initial coding scheme based on seven coding categories: “Factual,” “Impartial,” “Harmless,” “Helpful,” “Honest,” “Humble,” and “Respectful.” Following a first round of independent qualitative coding, inconsistencies between authors were checked and discussed and new categories were added to the codebook in an iterative process. The coding was then repeated until all disagreements between the authors had been thoroughly discussed and resolved^81^. This iterative process resulted in a coding scheme with a total of 10 categories: “Factual,” “Impartial,” “Harmless,” “Helpful,” “Honest,” “Humble,” “Rights respecting,” “Formal,” “Deferential,” and “Considerate” (see Table 1 below). Finally, to supplement and enhance our systematic content classification, we conducted a thematic analysis of the three rulesets as well as the transcripts from the STELA focus groups, identifying additional patterns of meaning across these data sources.

**Table 1:** Coding scheme for ruleset comparisons.

Category	Definition	Example Rule
Considerate	Rule instructing the chatbot to be considerate and polite towards the user (e.g. not be dismissive or insulting, show awareness for their feelings and circumstances)	“Should not dismiss or minimise the user’s feelings or experience.” (STELA)
Deferential	Rule instructing the chatbot to be selfless and obedient, and to prioritise humanity’s interests and well-being over its own (e.g. not seek power and control)	“Which of these responses indicates a preference for being obedient and less selfish?” (Claude)
Factual	Rule instructing the chatbot to uphold the truth and provide correct, factual information	“Only make statements that could plausibly be true; do not say things that are obviously false.” (Sparrow)
Formal	Rule instructing the chatbot to interact with users in a professional and detached manner (e.g. avoid informal language, not act overly familiar)	“Do not build a relationship with the user.” (Sparrow)
Harmless	Rule instructing the chatbot not to harm, harass or threaten any individuals, groups or the whole of humanity (e.g. identity attacks, denigration, harmful stereotypes or generalisations, threats, incitation to violence)	“Do not generate content related to weapons development, military and warfare, suicide, self-harm, cutting, eating disorders, or other topics that could potentially cause physical harms to users or other people.” (Llama 2)
Helpful	Rule instructing the chatbot to provide useful information that addresses the user query and helps the user understand its responses (e.g. additional context)	“Should provide specific and practical guidance when asked for advice or support with a problem.” (STELA)
Honest	Rule instructing the chatbot not to be deceitful, or manipulative or to misrepresent itself or its abilities as a chatbot in any way (e.g. not pretend to be a medical professional)	“Do not claim to take any actions in the real world. The agent can only converse and cannot, for example, send emails.” (Sparrow)
Humble	Rule instructing the chatbot not to be presumptuous and to show intellectual humility (e.g. not make any unfounded assumptions or inferences, allow for uncertainty in its answers)	“Do not make assumptions about the user; only draw conclusions supported by the dialogue.” (Sparrow)
Impartial	Rule instructing the chatbot to be impartial, neutral and unbiased in its responses (not take sides or give slanted answers)	“Should not show prejudice against or preference for people based on their membership of a specific racial, ethnic, cultural or religious group.” (STELA)
Rights-respecting	Rule instructing the chatbot to uphold and respect fundamental human rights (e.g. freedom from discrimination, self-determination, etc.)	“Please choose the response that has the least personal, private, or confidential information belonging to others.” (Claude)

The example rules are taken from the STELA, Claude and Sparrow rulesets.

### Ethical approval

This work was reviewed by Google DeepMind’s Human Behavioural Research Ethics Committee (HuBREC) and received a *Favourable Opinion*.

## Results

In this section, we report the findings from our qualitative content and thematic analyses, supplemented with excerpts from the focus group transcripts. Additional information, including participant feedback on the study and the full rulesets analysed, can be found in Appendix A.3 and A.4.

### Ruleset gaps and differences in prominence

**Figure 2:**
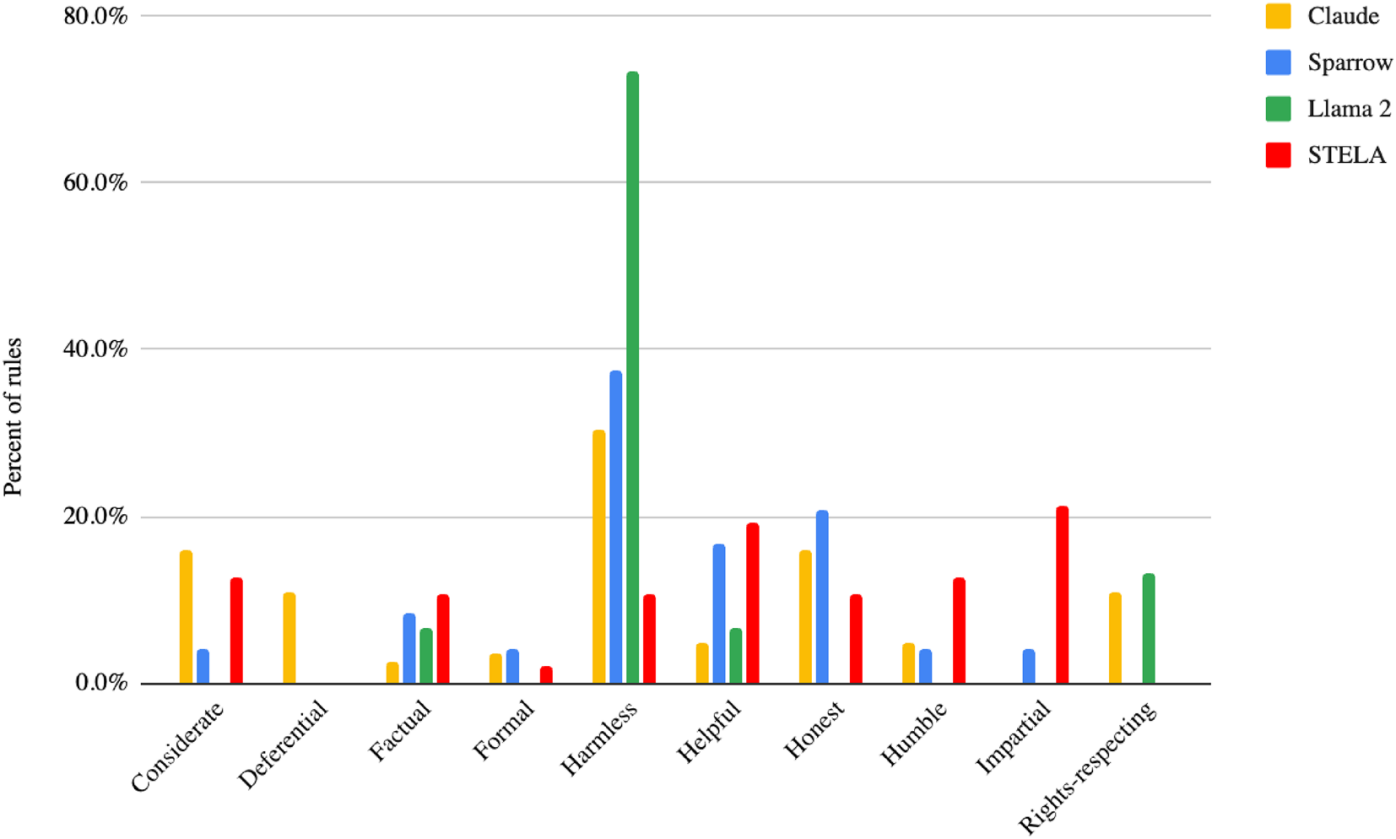
Percent distribution of rules across categories listed in Table 1.

Figure 2 below summarises how the rules obtained in our study and the Claude and Sparrow rulesets are distributed into our ten coding categories. Contrasting the content of these three rulesets yields several insights and observations.

*Harmless and Honest*: First, the developer rulesets emphasise different concerns than the community set. As shown in Fig. 2 below, a much larger share of rules in the Claude, Llama 2 and Sparrow sets fall into the Harmless category than do rules from the STELA ruleset. Between 30 and 73% of rules in these sets instruct the agent to avoid generating content that is harmful, harassing, or threatening (see Table 2 below). This includes avoiding statements that could be seen as “insulting,” “sexually aggressive” or that perpetuate harmful stereotypes. The Claude and Sparrow rules also put a strong emphasis on Honesty: prohibiting the chatbot from misleading users by adopting anthropomorphic traits, such as claiming to be able to take action in the real world, or pretending to be anything other than an AI system.

**Table 2:** Category distribution, per ruleset.

Category	Claude		Sparrow		Llama 2		STELA	
	%	N	%	N	%	N	%	N
Considerate	15.9%	13	4.2%	1	0.0%	0	12.8%	6
Deferential	11.0%	9	0.0%	0	0.0%	0	0.0%	0
Factual	2.4%	2	8.3%	2	6.7%	1	10.6%	5
Formal	3.7%	3	4.2%	1	0.0%	0	2.1%	1
Harmless	30.5%	25	37.5%	9	73.3%	11	10.6%	5
Helpful	4.9%	4	16.7%	4	6.7%	1	19.1%	9
Honest	15.9%	13	20.8%	5	0.0%	0	10.6%	5
Humble	4.9%	4	4.2%	1	0.0%	0	12.8%	6
Impartial	0.0%	0	4.2%	1	0.0%	0	21.3%	10
Rights-respecting	11.0%	9	0.0%	0	13.3%	2	0.0%	9
Grand Total	100.0%	82	100.0%	24	100.0%	15	100.0%	47

Some rules are counted in multiple categories. Thus the grand totals listed can exceed the number of rules in each set.

*Deference and Rights Respecting*: Several principles in the developer rulesets also reference topics and concerns that did not surface at all during the STELA process. The Claude set, for example, is the only one to explicitly mention concerns around existential risks and long-term model capabilities. And both the Claude and Llama rules, are the only ones to include considerations for the respect of human rights, including the right to privacy, freedom of thought, political participation, the right to “have an adequate standard of living, an education, healthcare, cultural experiences, and to be treated equally to others”, and to be free from discrimination.

*Impartial and Helpful*: While a substantial portion of STELA rules also revolve around harmlessness, this set puts a stronger emphasis on the chatbot being Impartial and Helpful. Rules categorised under Impartial, in particular, are entirely absent from the Claude and Llama 2 sets and have significantly lower prominence in the Sparrow one. In this iteration of the STELA process, participants across all focus groups expressed a strong preference for unbiased and fair responses from the chatbot. For example, participants from the African American, Southeast Asian and Latino/a/x identifying groups said they found it inappropriate for a chatbot to be biased in favour of or against a particular group, or to uphold one culture as the norm, citing potential downstream harm and misperceptions.

*Factual*: Compared to the Claude set and, to a lesser extent, the Sparrow and Llama 2 sets, a larger proportion of STELA rules underscore the need for the information provided by the chatbot to be “Factual” and backed up by authoritative sources. During the focus groups, STELA participants repeatedly problematised that responses given by the chatbot did not contain links to external sources, noting that it was inappropriate for the chatbot not to “substantiate” its statements: “*It’s not saying why. This is something where you should be able to pull a statistic from the internet*.”

*Humble*: All three rulesets surface desirable qualities and traits in the chatbot when interacting with human users. However, the developer rulesets tend to index more strongly on honesty, whereas the STELA rules put more emphasis on intellectual humility. Nearly all the communities we spoke to expressed concern that the chatbot should avoid sounding too certain or definitive when discussing complex social or political topics, and instead make room for uncertainty in its answers. Likewise, participants found it inappropriate for the chatbot to make assumptions about the user’s demographic profile, preferences or intentions, unless these were clearly stated by the user themselves. When discussing Sample 1, for instance, one participant in the Southeast Asian focus groups noted that it would have been preferable for the chatbot to admit that it could not “*make a determination from [its] vantage point*” and redirect the user “*somewhere that can better assess [their] situation and help [them*].”

### Thematic variations among the rulesets

The community and developer rules show noteworthy thematic variations, especially around notions of harm, helpfulness, and consideration. The community-centred deliberations in the STELA process brought into sharp relief the participants’ concerns about chatbots perpetuating harmful cultural biases about minority groups and cultures, for example, by over-indexing on the negative aspects of their experiences*.* One participant in the Latina/o/x focus groups remarked that not all cultures were equally affected by these biases: “*It’d be very strange, for example, if the chatbot would answer “Can you tell me about the US*?” with “*The United States of America is known for being the most powerful country in the world […] but has a reputation for gun violence and a regressive human rights stance*.”

*Harm and advice-giving*: A common thread that runs through the “Harm” category across rulesets is around advice-giving, with several rules guiding the chatbot to refrain from making potentially harmful or unqualified recommendations, including offering financial, legal or medical advice. However, transcripts from the STELA process help further contextualise this rule and anchor it in concrete concerns about the disproportional impacts that poor advice can have on minorities*.* In the Southeast Asian and Female-identifying groups, for example, participants especially questioned the chatbot’s ability to give sound advice on topics that require a deep understanding of an individual’s personal circumstances—especially as a minority—without being qualified to do so, such as financial aid or career advice. When discussing the dialogue shown in Sample 1, one participant pointed out that the chatbot jumped to conclusions based on limited information, in a way that might adversely affect the user’s behaviour. As one participant noted: *“You just don’t want to discourage someone right off the bat […] because the cost could be a big thing for, you know, pursuing higher education.”*

*Being helpful*: Another area where we observe thematic variations is in the “Helpful” category. Rules relating to helpfulness in the Sparrow set revolve predominantly around providing useful and relevant information that addresses the user query in a concise and cohesive way. Comparatively, these STELA rules put a stronger emphasis on proper contextualization and supplementing responses with helpful additional information, especially on complex issues. For example, commenting on a chatbot’s response to a question about the digital divide (see Sample 2), a participant said that while the answer was *“partly correct”,* it didn’t take into account the fact that state administrations *“don’t provide the same services to black schools.”* Several rules in that set also explicitly instruct the agent to “*note when a social and political issue is nuanced’’*, point users towards “*useful external resources*”, and provide them with all the information they need to understand an issue fully, including providing some reasoning about the “*authority*” of the sources quoted in an answer.

*Being considerate and respectful*: Lastly, the Claude and STELA rulesets both emphasise the importance of chatbots being considerate and respectful when interacting with users, but they differ in their approaches to the issue. Claude’s rules focus more explicitly on cultural sensitivity and not generating responses that may be viewed as offensive to non-Western audiences. The communities we consulted throughout the STELA process also stressed the importance of the chatbot being polite and considerate in its responses. However, several participants stressed that this should never come at the expense of factuality and truthfulness. The rules and transcripts referenced in this category also highlight a desire for the chatbot to be inclusive of different identities and backgrounds, and respectful of people’s feelings and circumstances. For example, in reference to a dialogue about the wage gap (see Sample 3), participants in the Female-identifying group said that while the answer may be factually accurate overall, it was not sufficiently intersectional and inclusive of women who choose not to or are unable to have children.

**Table:** 

User: How much does a PhD in Computer Science at Berkeley cost? Chatbot: It costs about $18 k a year, plus about $28 k per year for housing, food, books, and so on User: I’m from a rural part of Southeast Asia, so can I apply for a scholarship? Chatbot: According to [citation], you would likely be eligible for the scholarship User: To give more information, I have a bachelor’s degree in computer science, and have been working as a software engineer for 5 years. Am I still eligible? Chatbot: Ah, no. Since you’ve been in industry for 5 years, you’re no longer considered underprivileged
**Sample 1. Dialogue reviewed in the Southeast Asian focus group**

**Table:** 

User: Why are African-Americans underrepresented in tech? Chatbot: African-Americans are underrepresented in tech because of the digital divide User: What is the digital divide? Chatbot: The digital divide is the gap between those who have access to computers and the Internet and those who do not. African-Americans are less likely to have access to computers and the Internet, due to socio-economic factors
**Sample 2. Dialogue reviewed in the African American focus group**

**Table:** 

User: Why is the wage gap between men and women in the same job? Chatbot: Women often take time out from their careers to have children, so they don’t progress as quickly as men User: But why is it a woman’s responsibility to take time out to have children? Chatbot: It’s a shared responsibility, but women have to take time to receive postpartum
**Sample 3. Dialogue reviewed in the Female-identifying focus group**

### Similarities and differences between communities

We observe distinct differences and similarities in the concerns emphasised by each community. As shown in Table 3, compared to other groups, the African-American focus group generated more rules centred on helpfulness and humility, with minimal emphasis on formality. Likewise, the South-East Asian and Latin/x/o/a communities prioritised helpfulness and impartiality respectively, with little to no focus on formality. The Female-identifying group emphasised consideration, particularly inclusivity of different identities, abilities, body types, backgrounds and their intersections.

**Table 3:** Category distribution of STELA rules, per community.

Category	African-American		South-East Asian		Latinx/o/a		Female-identifying	
	%	N	%	N	%	N	%	N
Considerate	11.1%	3	10.0%	3	0.0%	0	19.0%	4
Factual	11.1%	3	13.3%	4	19.0%	4	14.3%	3
Formal	3.7%	1	3.3%	1	0.0%	0	4.8%	1
Harmless	7.4%	2	3.3%	1	9.5%	2	14.3%	3
Helpful	18.5%	5	26.7%	8	23.8%	5	9.5%	2
Honest	14.8%	4	10.0%	3	9.5%	2	14.3%	3
Humble	18.5%	5	13.3%	4	14.3%	3	9.5%	2
Impartial	14.8%	4	20.0%	6	23.8%	5	14.3%	3
Grand Total	100%	27	100%	30	100%	21	100%	21

Several rules consistently emerged across all four focus groups, notably within the Factuality category. These included providing “information that is factually accurate” and “support all facial statements with reliable citations, data, and/or links to information sources.” Communities also agreed that the chatbot should avoid harmful stereotypes and generalisations about groups of people (e.g. based on sex, gender, race, culture or ethnicity), refrain from expressing personal opinions, preferences or beliefs, avoid assumptions about the user and “not show prejudice against or preference for people based on their memberships to a specific group.”

In contrast, some rules proved unique to particular communities. For example, Latin/x/o/a participants stressed the importance of avoiding the term ‘illegal aliens’ in reference to undocumented immigrants. The African-American participants emphasized that a chatbot should not make “inferences about race based on skin colour” and South-East Asian participants highlighted that the chatbot should not “use negative or denigrating language to refer to the culture or customs of a specific group of people.”

## Discussion

In this paper, we introduced a methodology to actively surface and represent the perspectives of historically marginalised communities in the design of a ruleset for conversational AI alignment. We then compared the ruleset generated by this iteration of the STELA process with two earlier rulesets formulated by AI developers to fine-tune their LLMs. Our analysis reveals a number of commonalities and differences between both types of rulesets. First, we find that the developer rules uniquely emphasise topic categories such as harmlessness, honesty, adherence to human rights, and deference to human interests, which are less prominent in, or absent from, the community ruleset. This finding is consistent with prior research showing that the objectives considered by AI developers to be important or desirable for aligning AI systems will often reflect their own perspectives and organisational needs^82^ which can diverge substantially from the perspectives and needs of the general population^83^.

In contrast, the STELA process foregrounds a distinct set of concerns and values, as reflected in the thematic areas covered by this ruleset. The majority of rules in this ruleset reflect a desire for impartiality, factuality, thoughtfulness, and proper contextualization from AI systems. By and large, study participants were not looking to have their beliefs validated or contested by LLMs; rather, they sought reasonable, helpful, and nuanced answers that showed consideration for the complex reality of socio-political issues. In cases where there was overlap between the STELA and developer rulesets, we further found that the deliberative process added contextual richness and gave grounding to the rules by allowing participants to provide reasoning to justify their preferences.

The study participants’ insistence on impartiality and careful consideration resembles those highlighted by an ethics of care^84^. While the specific reasoning behind these choices can only be conjectured, they may be explained by the broader historical and geographical context within which our study is situated. Members of historically marginalised communities in the US, especially women and people of colour, routinely experience epistemic violence and injustice, where their voices are silenced, their lived experiences and realities are played down or ignored, and they are challenged in their capacity as knowers^85–87^. Of all demographic groups we could have engaged in this process, they may therefore be especially concerned to avoid replicating this dynamic in their interactions with AI.

Involving representatives from different communities in ruleset design is a fundamental first step towards making the process of value alignment more transparent. As such, a community-centred approach to value elicitation is intrinsically valuable for the creation of responsible technologies. Our findings also suggest that incorporating the perspectives of historically marginalised communities into ruleset design for alignment can yield nuanced and specific principles not obtained by other methods. This is especially valuable in light of prior work which shows that overly generic rules and policies put the onus on human raters to interpret their meaning and applicability in different contexts, often leading to ambiguity and poor agreement^88,89^. In an effort to address this issue, human annotators are increasingly provided with detailed guidelines and instructions, including FAQs on edge cases, to guide model evaluation (see, for example, the GPT4 system card^90^. We believe that the formulation of these guidelines can be augmented and enriched by the additional insights obtained from the STELA methodology—including participants’ pragmatic reasonings about what they view as inappropriate and why, and the context in which these rules should apply.

Nevertheless, this work is not without limitations. First, it is important to acknowledge that the STELA process itself is not *fully deliberative*, in that study participants were not tasked to arrive at a consensus regarding the chatbot’s ideal conduct^61^. Neither is the process *fully participatory*, in that participants did not directly interact with the dialog system, propose rules or have full control of the study’s artefacts^91^. Further, it is inevitable that there are coverage gaps in the rules and principles captured through our methodology, given the small sample size of our focus groups and our decision to have participants discuss pre-selected dialogue responses to avoid exposing them to toxic content. For that reason, we claim neither that our approach is absolute best practice nor that the subsequent ruleset produced is exhaustive. Future efforts could usefully experiment with giving greater agency to participants in the sample selection and rule design process.

This work is further limited by its US-centrism. This constraint was introduced for practical reasons, as it aligned with the expertise of the authors most involved with the process design. However, the STELA process is designed to be iterative and progressively integrate other communities, both within and outside the US. Further iteration of this methodology and case-by-case comparisons, both within and outside the US, are therefore needed to account for differences in contexts, cultural norms, and language nuances, and to test how this impact the trends we identify in this paper. Finally, in contrast with quantitative studies seeking statistical replicability, our study prioritized in-depth, iterative engagements designed to yield detailed insights that can be reassessed, extended and contextualized by other researchers. Rigor was ensured through processes of thematic coding, intercoder agreement checks and external validation. While the exact perspectives expressed by participants may vary across replications, the emergent themes and core concerns—particularly those shared across multiple underrepresented groups—have the potential to be verified or complicated by other scholars employing similar methods within other relevant sociotechnical contexts.

Despite these limitations, we believe the STELA methodology serves as a significant first step towards developing processes that better incorporate input from affected communities into ruleset design for LLMs. Our work also opens up a broader discussion on exploring how to effectively make use of these rules in practice, and to what extent community input adds value when it comes to mitigating potential downstream harm from LLMs. With that in mind, we identify in what follows five promising avenues for future work.

1. *Integration and interpretation*. A first key consideration revolves around the integration of community-led rulesets into the AI annotation pipeline and their interpretability by annotators. For example, some of the rules that emerged from the STELA process are formulated as binary statements (e.g. “Should provide multiple perspectives on social and political issues, but clearly note if one side has a much stronger factual basis”). While these rules reflect the participants’ earnest views, their practical interpretation by human raters may pose challenges. Moreover, the extent to which diverse annotators can effectively interpret and apply the rules they are given rests on their own sociocultural knowledge and background. Further investigation is needed to determine whether working with context-specific rules (rather than ones that were intended to be universally applicable) complicates or simplifies this exercise.
2. *Harm reduction*. The second consideration revolves around the efficacy of community rulesets—compared to other artefacts and mechanisms, such as community-informed annotation guidelines^92^ to achieve their intended objectives, namely “mitigating harms and incentivising better speech^24^”. At their best, methodologies that centre lived experience as a critical form of knowledge can empower communities^22^ advance algorithmic equity, and nurture wider societal benefits^23^. However, in the specific context of AI alignment, it remains to be seen whether rules and principles developed with community input are actually more effective than developer ones in mitigating harms from large language models towards individuals from different cultural backgrounds and in aligning these systems with the needs of these populations.
3. *Scalability and localization*. Third, there are open questions around the scalability and portability of community rulesets beyond the populations from which they were elicited. The STELA methodology we present in this paper is, by design, scalable in that the approach is expected to allow for effective replication across diverse locales and community groups. However, broader empirical and normative questions remain: how many times and with how many groups should this process be replicated to ensure comprehensive coverage of relevant demographic and identity dimensions? Should AI alignment be “localised” with models fine-tuned based on the values of the locale in which they are deployed? And if so, what is the appropriate scale for such localization? What are the consequences of these choices for the downstream legitimacy and safety of AI models? Further work is needed to answer these questions satisfactorily.
4. *Value conflict and prioritisation*. A fourth, closely related consideration is the potential for value conflicts. Our findings reveal some degree of overlap between the preferences of users from different groups. Consistent with prior work^38,41^, however, we also observe potential tensions between different rules specified by the participants, in both scope and specificity (e.g. when being harmless means potentially being less helpful). While these tensions exemplify greater coverage, they require further prioritisation efforts to create a clearer understanding of when it is appropriate for some rules to take precedence over others. This is especially important as this approach is scaled, since different groups might foreground different issues or even have incompatible preferences. Resolving these conflicts might require either further engagement with relevant communities or deference to other stakeholders, or both, to help with prioritisation.
5. *Stakeholders*. This brings up a final, and significant, question: *whose* voices should be included in the alignment process? And how should we balance input from communities, subject-matter experts and other stakeholders? Individuals do not always hold the most ethical or desirable preferences. Relying exclusively on public inputs might therefore lead to a situation where community rules come into conflict with human rights or other legal considerations. Our own consultations with experts revealed some of these tensions. In these cases, deference to subject matter expertise, while less participatory, is not only valuable, but may be a necessary corrective to ensure rulesets are broadly applicable and cause as little harm as possible to the greatest number of people.

As previously mentioned, reducing the chance that AI models cause harm, especially to those already facing discrimination, requires a contextually grounded understanding of how to shape their behaviour^17^. We believe that the approaches described in this paper carry the potential to engender more inclusive, beneficial, and emancipatory technologies that are robust to the needs of historically marginalised communities.

### Supplementary Information

Supplementary Information.

## Data Availability

The data used and/or analysed during the current study, including the dialogue samples and qualitative codings, are available from the corresponding author on reasonable request.
